# Hospital Meetings, &c.

**Published:** 1897-05-15

**Authors:** 


### HOSPITAL MEETINGS, &C.

### GREAT NORTHERN CENTRAL HOSPITAL.

Mr. Benjamin L. Cohen, M.P., who presided at the festival dinner of the Great Northern Central Hospital, which took place on Tuesday night, the 11th inst., at the Hotel Cecil, set himself the task of proving that the hospital satisfied the two conditions necessary to ensure the support of the district to which it ministers, viz.: first, that the hospital deserved support; and secondly, that it needed it. The claims of the hospital, he said, were many. First, it was the only hospital in Islington, with a population of something like 340,000, and it therefore had a paramount claim on the inhabitants of that district; but, more than this, it was practically the only hospital for the whole of North London, no less than 40 per cent. of the patients coming from Hampstead, Highgate, Barnet, Finchley, Tottenham, Wood Green, and Hornsey. Therefore, although the population of the parish of Islington was only 340,000, it might well be said that the hospital ministered to the wants of nearly a million of human beings. That brought him to the second condition he had set out to prove—that help was needed—and he proceeded to show first that the provision made by the hospital was totally inadequate; and secondly, that the income received was more inadequate still. The hospital at most could only provide 155 beds for the accommodation of the huge mass of human beings in North London, but of these at the present time the committee could only afford to furnish and keep up 107, two wards (with room for 48 beds) never having been opened owing to lack of funds. The consequence of this was that hundreds of patients had to be turned away yearly for want of accommodation. The income from endowments was not sufficient to pay the expenses of the nursing staff alone, while their income from fairly reliable sources, such as annual subscribers, Hospital Sunday and Saturday Funds, &c., only came to about £5,600 out of the £9,000 which is required yearly to keep the 107 beds open. Mr. Cohen also in the course of his speech said that Islington did not do its duty by the hospital. It sent to the hospital nearly 60 per cent. of the patients who received treatment, while it subscribed less than 10 per cent. of the total income.—Mr. C. T. Murdoch, M.P., chairman of the hospital, responded.—The dinner resulted in a contribution list of nearly £3,200.

### EAST LONDON HOSPITAL FOR CHILDREN.

The East London Hospital for Children also held a festival dinner at the Hotel Cecil on Tuesday, the 11th inst. Lord Herschell, G.C.B., presided, and amongst those present were Lady Herschell, Lord and Lady Falkland, Lady Helen Clifford Mellor, the Bishop of Stepney, Mr. W. R. Bousfield, Q.C., M.P., Mr. Michael Biddulph, M.P., Major and Mrs. J. Roper Parkington, Mr. Charles Cheston, Mr. H. J. Alcroft, and Mr. H. W. Trinder. The Chairman, in proposing the toast of the evening, "The East London Hospital for Children," said that without going into statistics he might yet mention as a proof of the necessity for this institution that in 1896, although some of the wards were closed for months, 1,500 in and 35,000 out-patients received treatment at the hospital. The hospital needed, in addition to increased ordinary income, a sum of £1,900 to pay for recent improvements.—The Bishop of Stepney, in proposing the toast of "The Board of Management," expressed his gratification at the fact that the hospital reserved two-thirds of its legacies for investment, and only spent one-third of them for ordinary expenses.—Subscriptions and donations amounting to £1,027 were announced during the course of the evening.

### NEW HOME FOR EPILEPTICS.

On Thursday, May 6th, the foundation-stone of a new Home for Men was laid at the Colony for Epileptics at Chalfont St. Peter, Bucks, by the Hon. T. Bayard, the late Ambassador of the United States. At the same time Mrs. Bayard opened "Eleanor House," the newly-completed Home for Women. Mr. Passmore Edwards has been the generous benefactor to the colony in both instances, the new men's home being given by him in special commemoration of the Queen's long reign, and named "Victoria House" in honour of the occasion. Mr. and Mrs. Bayard, with Mr. and Mrs. Passmore Edwards, Sir William Broadbent, Sir James Crichton Browne, Mr. F. D. Mocatta, Mr. E. Montefiore Micholls chairman of the Society for the Employment of Epileptics, with many others travelled down to Chorley Wood by special train. At the colony a large marquee was erected on the site of the new building, and here a short dedicatory service was conducted by the Vicar of Chalfont St. Peter, after which Mr. Micholls said a few words on the objects of the Society for the Employment of Epileptics. The foundation-stone was then laid by Mr. Bayard, who took the opportunity to comment upon this most memorable year in the history of the British Empire, and to remark that it was well to commemorate this year of Jubilee by such a work as that which had brought them together, and to give it the name of Queen Victoria. The Queen wished for no personal admiration nor personal gain, but gave a keynote that marked the year with charity and loving-kindness among all her people. It was with gratitude that, after four years of service an envoy from his country to this, he found it still his privilege to speak on such an occasion. It was sought to make this home the scene of health, where sufferers should regain the equilibrium of their faculties by healthful exercise in a healthful atmosphere. It was not well that all the strength of a country should belong to a single class, but there should be a healthful flow of mutual interest and activity in all classes. Such mutual interest existed in this community, and their devotion, charity, and voluntary benevolence were most honourable to British subjects. Sir James Crichton Browne finally moved a vote of thanks to Mr. and Mrs. Bayard for their presence, and to Mr. Passmore Edwards for his munificent gifts to the Colony, the visitors afterwards going over to the women's house, which was formally declared open by Mrs. Bayard.

